# Behavioural benefits of multisensory processing in ferrets

**DOI:** 10.1111/ejn.13440

**Published:** 2016-11-03

**Authors:** Amy Hammond‐Kenny, Victoria M. Bajo, Andrew J. King, Fernando R. Nodal

**Affiliations:** ^1^ Department of Physiology, Anatomy and GeneticsUniversity of OxfordOxfordOX1 3PTUK

**Keywords:** auditory localization, multisensory integration, orienting, reaction time, visual localization

## Abstract

Enhanced detection and discrimination, along with faster reaction times, are the most typical behavioural manifestations of the brain's capacity to integrate multisensory signals arising from the same object. In this study, we examined whether multisensory behavioural gains are observable across different components of the localization response that are potentially under the command of distinct brain regions. We measured the ability of ferrets to localize unisensory (auditory or visual) and spatiotemporally coincident auditory–visual stimuli of different durations that were presented from one of seven locations spanning the frontal hemifield. During the localization task, we recorded the head movements made following stimulus presentation, as a metric for assessing the initial orienting response of the ferrets, as well as the subsequent choice of which target location to approach to receive a reward. Head‐orienting responses to auditory–visual stimuli were more accurate and faster than those made to visual but not auditory targets, suggesting that these movements were guided principally by sound alone. In contrast, approach‐to‐target localization responses were more accurate and faster to spatially congruent auditory–visual stimuli throughout the frontal hemifield than to either visual or auditory stimuli alone. Race model inequality analysis of head‐orienting reaction times and approach‐to‐target response times indicates that different processes, probability summation and neural integration, respectively, are likely to be responsible for the effects of multisensory stimulation on these two measures of localization behaviour.

## Introduction

Perception of events in a natural environment typically depends on multiple facets of information derived from different forms of energy (e.g. sound pressure waves and electromagnetic radiation), with each having specific transduction requirements and encoded within different neural circuits. However, to generate a unified percept, these circuits must converge and their information must be integrated. This process of multisensory integration is important not only for scene analysis but has been shown, by reducing perceptual ambiguity, to confer a range of behavioural advantages from enhanced detection to improved object recognition (reviewed in Murray & Wallace, 2012).

The capacity to merge different sensory signals from the same region of space is thought to result in faster and more accurate responses and is one of the key factors determining how those signals are integrated in the brain (Stein & Stanford, 2008). Vision and hearing are generally the most important sensory modalities for perceiving distant objects, with the former providing high‐resolution spatial information, whereas the latter can be used to localize objects and events even if they are not visible. However, combining spatial information across these sensory modalities is challenging because visual and auditory signals are encoded in different ways within their respective sense organs and brain pathways. Positions in visual space are represented by the locus of activity within topographic projections from the retina, whereas the auditory system is organized tonotopically, which means that the location of a sound source has to be derived from monaural and binaural spatial cues that arise from the geometry of the head and external ears (King *et al*., 2001).

A range of behavioural tasks have been used to measure the effects of auditory–visual interactions on localization behaviour. These include saccadic eye movements with the head still (e.g. Frens *et al*., 1995; Frens & Van Opstal, 1998; Harrington & Peck, 1998; Colonius & Arndt, 2001; Corneil *et al*., 2002), combined eye and head gaze shifts (Goldring *et al*., 1996), head movements (Ho *et al*., 2013) and manual responses (Alais & Burr, 2004; Odegaard *et al*., 2015). However, these studies have produced variable results in terms of whether combined auditory–visual stimuli actually result in faster and more accurate responses than those elicited by the constituent unimodal sensory stimuli. This variation at least in part reflects differences in the complexity and type of stimuli used and in the requirements of the task.

In this study, we measured the timing, accuracy and precision of two measures of localization behaviour to assess the ability of ferrets to integrate spatiotemporally coincident visual and auditory cues. Ferrets have been used extensively in physiological and anatomical studies of multisensory processing (e.g. King & Hutchings, 1987; King *et al*., 1988; King & Schnupp, 2000; Bizley *et al*., 2007; Bizley & King, 2008; Stitt *et al*., 2015), but only to a limited degree so far in behavioural experiments (Isaiah *et al*., 2014; Hollensteiner *et al*., 2015), despite the ease with which they can be trained to carry out localization and other sensory tasks. We have previously characterized sound localization behaviour in this species by measuring their head‐orienting response following stimulus presentation and the subsequent locomotor response as the animals approach the perceived location of the sound source to receive a water reward (Nodal *et al*., 2008). Although both measures are part of the natural orienting response of the animals, they are differentially affected by lesions (Nodal *et al*., 2010) or reversible deactivation (Nodal *et al*., 2012) of the auditory cortex, implying that different neural circuits are involved in guiding accurate head‐orienting and approach‐to‐target behaviour.

Our results show that the integration of spatiotemporally coincident auditory and visual cues results in significantly faster and more accurate approach‐to‐target responses throughout the frontal hemifield. In contrast, for the head movements, this auditory–visual advantage was observed relative to visual but not auditory targets only, implying that a neural integration stage is not required to account for the multisensory effects on the animals’ initial orienting responses.

## Materials and methods

All procedures using animals were approved by the University of Oxford Animal Care and Ethical Review Committee and performed under licence from the UK Home Office in accordance with the Animals Scientific Procedures Act (1986, 2012).

### Animals and welfare

Five adult female sable‐coated ferrets (*Mustela putorius furo,* age at training onset: 6–24 months) were used in this study. Animals were housed in groups of up to three in standard laboratory cages (L × W × H: 76.2 × 76.2 × 86.4 cm) and maintained under controlled ambient conditions that varied according to British Summer Time (summer: 15 : 9 h light/dark cycle and 21–24 °C; winter: 8 : 16 h light/dark cycle and 17–20 °C). The cage environment was enriched with objects such as balls, tubes and shelters. Prior to starting the task, otoscopic examination and tympanometry were performed on each animal to exclude any abnormalities of the outer and middle ear.

During behavioural testing periods, which each lasted for five consecutive days, animals were motivated to perform the task by regulating their access to water. In these testing periods, *ad libitum* access to dry food was provided, whereas access to water was provided only during the twice‐daily testing sessions in the apparatus described below. If the total daily volume consumed during these testing sessions was < 60 mL/kg, the typical volume consumed when ferrets have free access to water, supplementary water was provided in the form of a mash comprising ground food pellets and sufficient water to make up the deficit. Body weights were measured on a daily basis and compared to individual baseline weights recorded at the start of each testing period. In the case of an animal losing > 15% of their baseline weight, which happened very infrequently, water regulation was stopped until its body weight recovered. Each testing period was followed by a break of at least 2 days during which the animals were provided with free access to water.

### Apparatus and stimuli

**Figure 1:**
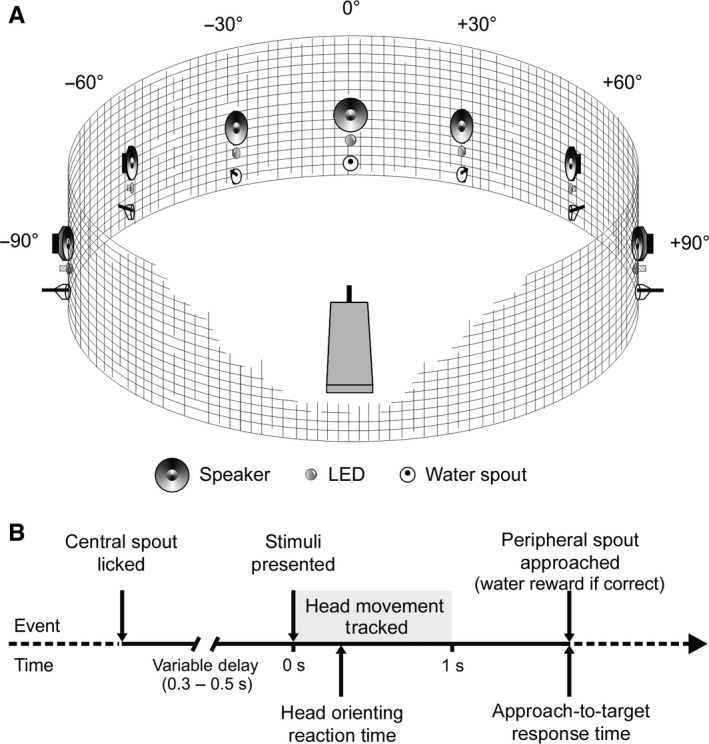
Behavioural Task Schematic. (A) Diagram of the behavioural testing apparatus. Auditory, visual or auditory–visual stimuli were presented from one of seven loudspeaker–LED pairs located at 30° intervals around the frontal perimeter of a circular arena (radius 70 cm). Correct approach‐to‐target responses were rewarded with water provided from a spout located underneath each loudspeaker–LED pair. Negative and positive angles denote the locations of loudspeaker–LED–spout combinations to the left and right of the midline, respectively. (B) Schematic representation of the sequence and timing of a single trial.

The localization task was performed in a custom‐built circular arena (70 cm radius) housed in a dimly illuminated (11.8 lx) sound‐attenuated chamber (Fig. 1). Animals were monitored from outside the chamber via a closed‐circuit TV monitor. To initiate a trial, animals were required to stand on a central raised platform and nose poke at the central waterspout, thereby ensuring they were facing straight ahead (defined as 0° location) when the stimulus was presented. Stimuli were presented from one of seven loudspeaker – light emitting diode (LED) pairs (loudspeaker: FRS 8, Visaton, Crewe, UK; LED: LTW‐2S3D8, Lite‐On, Milpitas, CA), positioned at 30° intervals in the horizontal plane around the perimeter of the frontal hemifield. A fixed water reward (typically 150–200 μL) was provided if animals correctly localized the stimulus by approaching and licking a waterspout positioned below each loudspeaker – LED pair. The first spout licked (approach‐to‐target response) and the time between the stimulus onset and this response (the ‘response time’) were recorded. Stimulus presentation, response registration and reward delivery were each controlled by a personal computer communicating with a System 3 TDT RX8 multi I/O processor (Tucker‐Davis Technologies, Alachua, FL) at a sampling rate of 100 kHz, using custom written scripts implemented in MATLAB (MathWorks, Natick, MA).

Stimuli comprised three types: auditory alone, visual alone and combined auditory–visual. Auditory stimuli consisted of single presentations of broadband noise bursts (with a low‐pass cut‐off frequency of 30 kHz) that were generated *de novo* on each trial. To disrupt ‘absolute level cues’ arising from acoustic shadowing by the animal's body and thereby prevent localization based on the relative loudness of stimuli, sound levels were roved pseudo‐randomly across trials from 56 to 84 dB SPL in 7 dB steps. In addition, to prevent localization based on spectral differences caused by the use of different loudspeakers, auditory stimuli were spectrally matched by convolving the signal with the respective loudspeaker's transfer filter. Visual stimuli consisted of illumination of a translucent plastic dome (2.5 cm diameter), positioned immediately below each loudspeaker and 10 cm above floor level, with a white light LED of 17 cd intensity and 15° viewing angle. During multisensory trials, spatially congruent auditory and visual stimuli were presented simultaneously.

### Head‐orienting responses

In addition to the approach‐to‐target responses, the change in the animal's head position was recorded for the first second following stimulus presentation. Head‐orienting responses were measured by tracking the *x*–*y* coordinates of an adhesive reflective strip attached to the midline of the scalp at a rate of 60 frames per second, using an overhead infrared‐sensitive camera (DMK 21BF04, The Imaging Source GmbH, Bremen, Germany). From these *x*–*y* co‐ordinates the head‐orienting reaction time and final bearing relative to the initial head position were derived using custom written scripts implemented in MATLAB.

The start of the head‐orienting response was defined as a movement in the same direction over three consecutive frames, with the timing of the first of these three frames relative to stimulus onset taken as the reaction time. The saccade‐like head movement was considered complete once a change in direction was detected. The final head bearing was taken as the mean angle from the last three frames of this movement or, if no change in direction was detected, as the mean angle from the last three frames recorded. Head‐orienting data were excluded from the subsequent analysis if the head bearing at stimulus presentation deviated by > 30° from the midline or if the reaction time was > 500 ms.

### Training and general procedure

Naïve animals took ~ 1 week to learn the behavioural procedure. During procedural training, animals were taught to stand on the platform and lick the central waterspout for a reward. Over the course of training, the probability of this reward was gradually reduced to 1/20. Once animals reliably positioned themselves correctly on the central platform, central spout contact was followed (after a variable delay of 300–500 ms) by continuous auditory–visual stimulus presentation from one of the seven pseudo‐randomly chosen locations. A peripheral reward was provided only if the animals correctly approached the location from which the stimulus was presented. All procedural training was carried out using auditory–visual stimuli to avoid introducing a training bias towards either sensory modality.

Once familiar with this procedure, the ability of animals to localize auditory–visual, auditory and visual stimuli, respectively, was tested in three separate blocks, to maximize the number of trials for each stimulus condition and ensure stable performance. Prior to starting each block, animals were familiarized with the stimulus condition until they showed no improvement in performance over five consecutive sessions (mean ± SE score for 2000 ms duration stimuli: auditory–visual (AV), 97 ± 1%; auditory (A), 92 ± 3%; visual (V), 83.8 ± 3%). Once criterion performance was achieved, behavioural testing was commenced and stimulus duration was progressively decreased in six steps (1000, 500, 200, 100, 40 and 20 ms), following the completion of at least 210 trials at each of the stimulus durations. To avoid a bias towards any stimulus location, incorrect responses were followed by at least one correction trial (same stimulus presented from the same location) and further incorrect responses were followed by up to two easy trials (continuous stimulus presented from the same location). Data from correction, easy and centre‐rewarded trials were not included in the analysis.

### Data analysis

All data were analysed using MATLAB. The results presented are based on the analysis of 22 826 approach‐to‐target responses, of which 18 267 trials yielded a head‐orienting trace. For the analysis, data were pooled across sound levels (56–84 dB SPL), as previous studies of auditory localization in the ferret have identified no difference in head‐orienting or approach‐to‐target performance across this range (Nodal *et al*., 2008). Head‐orienting performance was measured by calculating final head bearings and absolute error magnitudes (the angular difference between the final head bearing and the stimulus location). Similarly, approach‐to‐target performance was measured by calculation of percentage correct scores (number of correct trials/(number of correct trials + number of incorrect trials) × 100) and error magnitudes (the unsigned differences between the target and response locations). The mutual information (MI) between the approach‐to‐target response location or final head bearing and the target location for the different stimulus durations and types was calculated using the formula:

$$\text{MI}\left(r,s\right)=\sum_{r,s}p\left(r,s\right)\times \log_{2}\left[p\left(r,\;s\right)÷p\left(r\right)\cdot p\left(s\right)\right]$$

where *r* is the response location or final head bearing (binned in 7.5° bins), *s* is the target location, MI(*r*; *s*) is the MI between *r* and *s*, *p*(*r*, *s*) is the joint probability of *r* and *s* and is equivalent to *p*(*r*|*s*) *p*(*s*), where *p*(*s*) and *p*(*r*) are obtained from the overall distribution of target locations and response (either approach to target or head bearing) locations, respectively.

To quantify multisensory enhancement effects, percentage gain was calculated using the equation (multisensory performance–unisensory performance)/(unisensory performance) × 100). Mean and standard error of the mean were used to summarize data unless otherwise specified.

Head‐orienting reaction times and approach‐to‐target response times were also analysed. An important behavioural manifestation of multisensory integration is the speeding up of responses, a phenomenon known as the redundant signal effect (RSE; Todd, 1912). This can be accounted for by two classes of model: probability summation (race model; Raab, 1962) and signal integration (co‐activation model; Miller, 1982). The race model assumes that signals from different sensory modalities are processed within entirely independent channels, which compete for response initiation. Consequently, the RSE occurs as the likelihood of a faster time is greater when signals are available in more than one channel than with one alone. In contrast, the co‐activation model accounts for the RSE in terms of energy summation, whereby signal integration across channels results in the threshold for response initiation being reached more quickly.

To determine the basis for the RSE, reaction and response times were subjected to race model inequality analysis, whereby the observed RSE is compared with the maximum facilitation expected given a race scenario. The latter can be derived from the unisensory times using probability summation, corrected for the assumption of independence between the unisensory channels by inclusion of a product term in the following equation (Stevenson *et al*., 2014). If the observed RSE exceeds that predicted by probability summation then the race model is rejected and instead the existence of an integration mechanism is implied. For each animal, response times were pooled across each stimulus type and divided into 5% quantiles expanding from 5% to 95% of the distribution. At each quantile, an upper limit was placed on the cumulative probability of times predicted by the race model (CP_(t)Model_) using the equation:

$${\text{CP}}_{(t)\mathrm{Model}}=\left({\text{CP}}_{(t)A}+{\text{CP}}_{(t)V}\right)-\left({\text{CP}}_{(t)A}\times {\text{CP}}_{(t)V}\right)$$

where CP_(t)A_ and CP_(t)V_ are the cumulative probability of times for trials with auditory and visual stimuli, respectively. Assuming the race model, Miller's inequality would hold (i.e. CP_(t)AV_ – CP_(t)Model_ < 0). If at any quantile, the observed cumulative probability of times for trials with auditory–visual stimuli (CP_(t)AV_) exceeds the CP_(t)Model_ value (i.e. CP_(t)AV_ – CP_(t)Model_ > 0), then the model can be rejected.

### Statistical analysis

The effects of stimulus modality, duration and location on final head bearing errors were investigated by fitting a linear mixed effects model to the data in R (www.r-project.org). To reduce dimensionality, stimulus durations were collapsed into three categories: short (20–40 ms), mid (100–200 ms) and long (500–1000 ms). Shapiro–Wilk test and normal QQ plots both confirmed the goodness of the model fit by showing that the residuals were normally distributed. Similarly, the effects of stimulus modality, duration and location on the correctness of approach‐to‐target responses were examined by fitting a generalized linear mixed model for Bernoulli‐distributed responses to the data. A Monte–Carlo simulation of the fitted model with 20 samples was performed to check the model fit. Half‐normal QQ plots of the Pearson residuals for the observed and simulated data were then compared and showed a good match, therefore verifying the fit (for method see Collett, 2002). Differences between groups are expressed as the odds ratio (OR).

For both analyses, head bearing errors and percentage correct scores from the approach‐to‐target responses, a mixed model was required to allow for within‐ferret correlation by fitting a random effect across ferrets. In addition, head‐orienting reaction and approach‐to‐target response times were examined for effects of stimulus modality and duration by submitting individual trials to separate analyses of variance (anova), with ferret identity included as a factor. Significant results were followed up using Tukey's multiple comparison tests. To assess the significance of race model violations across individual animals, separate one‐sided *t*‐tests comparing the observed and modelled CP values were performed on each 5% quantile that exhibited a group average violation of the model.

## Results

To characterize fully the ability of ferrets to localize stimuli of different modalities (visual, auditory, and auditory–visual), the accuracy, precision and timing of their stimulus‐evoked head‐orienting movements and approach‐to‐target responses were recorded. Although each behaviour can be considered as a component of the localization response, there are fundamental differences between them, both in terms of the way they are affected by different stimulus parameters and the likely neural circuitry involved. Moreover, the head‐orienting responses, which were not shaped during the operant training, provide a continuous measure of localization performance, whereas the conditioned approach‐to‐target responses represent a categorical measure due to the discrete number of stimulus–response location combinations.

### Localization accuracy varies with the properties of the stimulus

**Figure 2:**
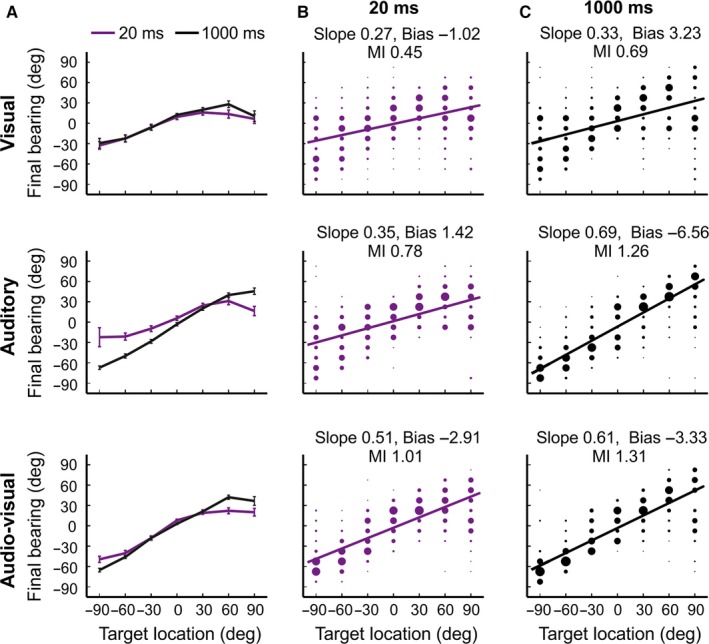
Effects of stimulus modality, duration and location on final head bearings. Distribution of final head bearings for each target location across different modalities (top row visual, middle row auditory and bottom row combined auditory–visual) and durations (20 and 1000 ms). (A) Mean final head bearings (± SEM) across each target location for 20 (purple) and 1000 ms (black) stimuli. (B, C) Confusion matrices for these data illustrating the distribution of final head bearings (bin size 7.5°) as a function of target location. For each stimulus location, the size of the dots is proportional to the probability of responses of different amplitudes. A regression line was fitted (purple and black lines) to each confusion matrix, using the linear least squares method, and the corresponding slope (gain) and y‐intercept (bias) are shown along with the mutual information (MI) values above the panels (see Table 1 for a complete set of values). Perfect performance equates to a gain of 1 and a response bias of 0. In general, final head bearings vary less with target location for visual stimuli than for auditory or auditory–visual stimuli. [Colour figure can be viewed at wileyonlinelibrary.com].

On most trials, stereotyped head‐orienting responses were elicited within 150 ms of stimulus onset. For all stimulus types, the amplitude of these movements co‐varied with the eccentricity of the target location, indicating that the animals turned their heads in the direction of the appropriate loudspeaker and/or LED prior to leaving the central platform (Fig. 2). On some trials, the final head bearing matched the target location, but, more commonly, it was smaller and therefore under‐shot the target (Fig. 2A–C).

Data were analysed by calculating the MI values and fitting a linear regression model to the final head bearing values vs. target locations (*R* ^2^ range across stimulus durations; visual: 0.17–0.35; auditory: 0.36–0.77; auditory–visual: 0.53–0.71) and the slopes and y‐intercepts of these lines compared across stimulus modalities and durations. The above parameters provide a measure of the relative accuracy of the head‐orienting response, with perfect performance equating to a slope (gain) of 1, a y‐intercept (response bias) of 0 and a theoretical maximum MI value of 2.8 bits. For all stimulus types, and particularly the auditory‐alone condition, increasing stimulus duration tended to result in an increase in the slope of these linear fits and greater MI values (Table 1). This was also associated with more precise responses, as indicated by less variation in the final head bearings at longer stimulus durations, particularly for the more eccentric target locations (> 30°).

**Table 1:** Linear model parameters and mutual information values for head‐orienting reactions and approach‐to‐target responses

	Head Orienting			Approach to Target		
	V	A	AV	V	A	AV
20 ms	0.27 (−1.02)	0.35 (1.42)	0.51 (−2.91)	0.54 (−7.63)	0.85 (4.11)	0.88 (0.86)
	0.45	0.78	1.01	0.47	1.36	1.51
40 ms	0.29 (−2.86)	0.49 (0.40)	0.51 (−4.53)	0.45 (−12.4)	0.82 (0.82)	0.85 (2.49)
	0.49	0.97	0.98	0.50	1.32	1.54
100 ms	0.28 (0.28)	0.47 (−2.30)	0.49 (−3.00)	0.50 (−11.1)	0.84 (−0.80)	0.88 (−0.72)
	0.45	0.87	0.91	0.59	1.33	1.67
200 ms	0.26 (−10.1)	0.53 (−2.04)	0.47 (−2.24)	0.42 (−10.2)	0.82 (−0.83)	0.90 (−1.74)
	0.52	1.02	0.95	0.60	1.43	1.88
500 ms	0.39 (−3.15)	0.53 (−0.12)	0.62 (−5.55)	0.70 (−3.40)	0.87 (−0.80)	0.93 (−2.40)
	0.69	1.15	1.35	1.20	1.78	2.23
1000 ms	0.33 (3.23)	0.69 (−6.56)	0.61 (−3.33)	0.73 (−4.07)	0.93 (−1.09)	0.96 (−0.23)
	0.69	1.26	1.31	1.57	2.05	2.39

Regression lines were fitted to plots of final head bearing values and approach‐to‐target response locations vs. target locations. The slopes and y‐intercepts (in parentheses) of these regression lines are shown on the top line with the mutual information between response and target locations (in bits) below for each stimulus duration (rows, 20–1000 ms) and type [columns, visual (V), auditory (A), and auditory–visual (AV)].

The lowest slopes were observed with visual stimuli, indicating that the animals made smaller head turns compared to the other stimulus conditions. However, if the most eccentric target locations (±90°) are omitted, then the linear regression fits improve for the responses to visual stimuli (*R* ^2^ range: 0.20–0.46) and the slopes (20 ms: 0.34; 40 ms: 0.41; 100 ms: 0.38; 200 ms: 0.35; 500 ms: 0.55; 1000 ms: 0.48) now resemble those observed for the auditory and auditory–visual condition. This suggests a similar involvement of head movements in the orienting behaviour of ferrets for all three stimulus types within the frontal quadrant, corresponding to the 90° wide binocular field of view in this species (Garipis & Hoffmann, 2003). The slopes of the regression fits for the relationship between target location and final head bearing were very similar for the auditory and auditory–visual stimuli, implying that the head‐orienting response is driven mainly by auditory stimuli, at least for the most eccentric locations.

**Figure 3:**
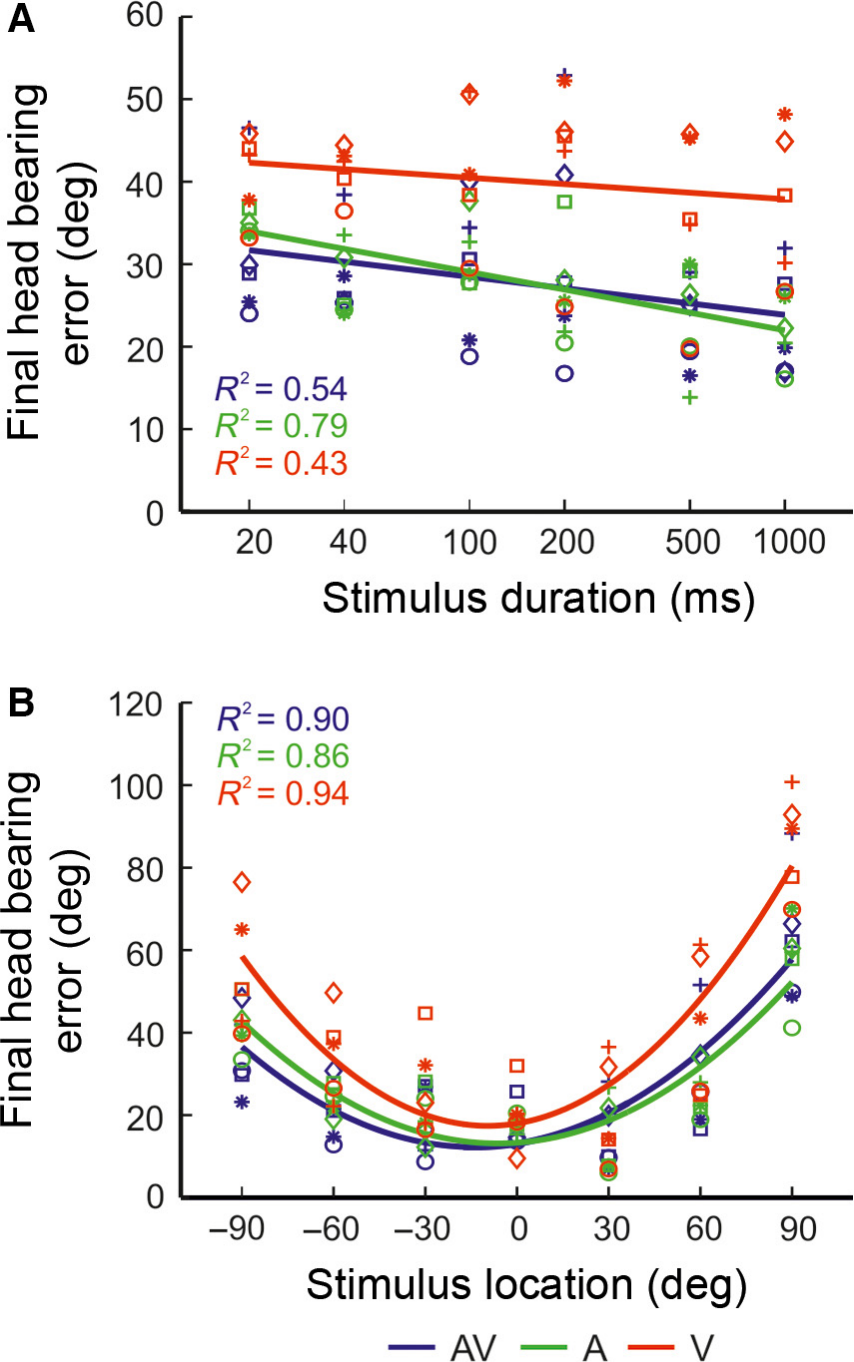
Comparison of head‐orienting accuracy to unisensory and multisensory targets. Head‐orienting errors (target location minus final head bearing) are shown as a function of stimulus duration (A), plotted on a log scale, and stimulus location (B) for auditory–visual (AV, blue), auditory (A, green) and visual (V, red) targets. Mean final head bearing errors were fitted with a regression line (A) or a polynomial function (B). The relevant co‐efficient of determination (*R* ^2^) value is shown for each dataset. Data from individual animals are represented by different symbols. Note that head‐orienting accuracy was similar for the auditory–visual and auditory targets across all stimulus durations and locations. [Colour figure can be viewed at wileyonlinelibrary.com].

To explore the effect of stimulus modality on head‐orienting responses, we calculated the final head bearing errors, i.e. the difference between the final head bearing and the target location (Fig. 3). Overall, head‐orienting accuracy was greatest for the auditory–visual condition, as demonstrated by the smallest error magnitude (visual = 38.5 ± 0.35°; auditory = 27.2 ± 0.38°, auditory–visual = 26.4 ± 0.35°). Across all stimulus modalities, smaller error magnitudes were observed at longer stimulus durations (Fig. 3A) and more central locations (Fig. 3B). A linear mixed effects analysis was performed to examine further the effects of stimulus modality, duration and location on final head bearing errors. With the exception of the 0° and ± 30° stimulus locations, the results show that final head bearing errors were significantly smaller for auditory–visual than visual stimuli (significance level range across stimulus locations by stimulus duration; 20–40 ms: *t*_(15792)_ = 4.82–11.48, *P *<* *0.001; 100–200 ms: *t*_(15792)_ = 3.25–9.94, *P* <* *0.001; 500–1000 ms: *t*_(15792)_ = 6.29–13.06, *P* < 0.001). In contrast, with the exception of a few stimulus combinations, 20–1000 ms at −90° and 20–40 ms at −60°, no significant difference in final head bearing error magnitude was identified between auditory–visual and auditory stimuli (20–40 ms: *t*_(15792)_ = 0.42–2.54, *P *>* *0.001; 100–200 ms: *t*_(15792)_ = −3.22–0.58, *P* >* *0.001; 500–1000 ms: *t*_(15792)_ = −2.52–1.28, *P *>* *0.001). This finding again suggests that, under multisensory conditions, the head‐orienting response may be driven primarily by the auditory modality.

**Figure 4:**
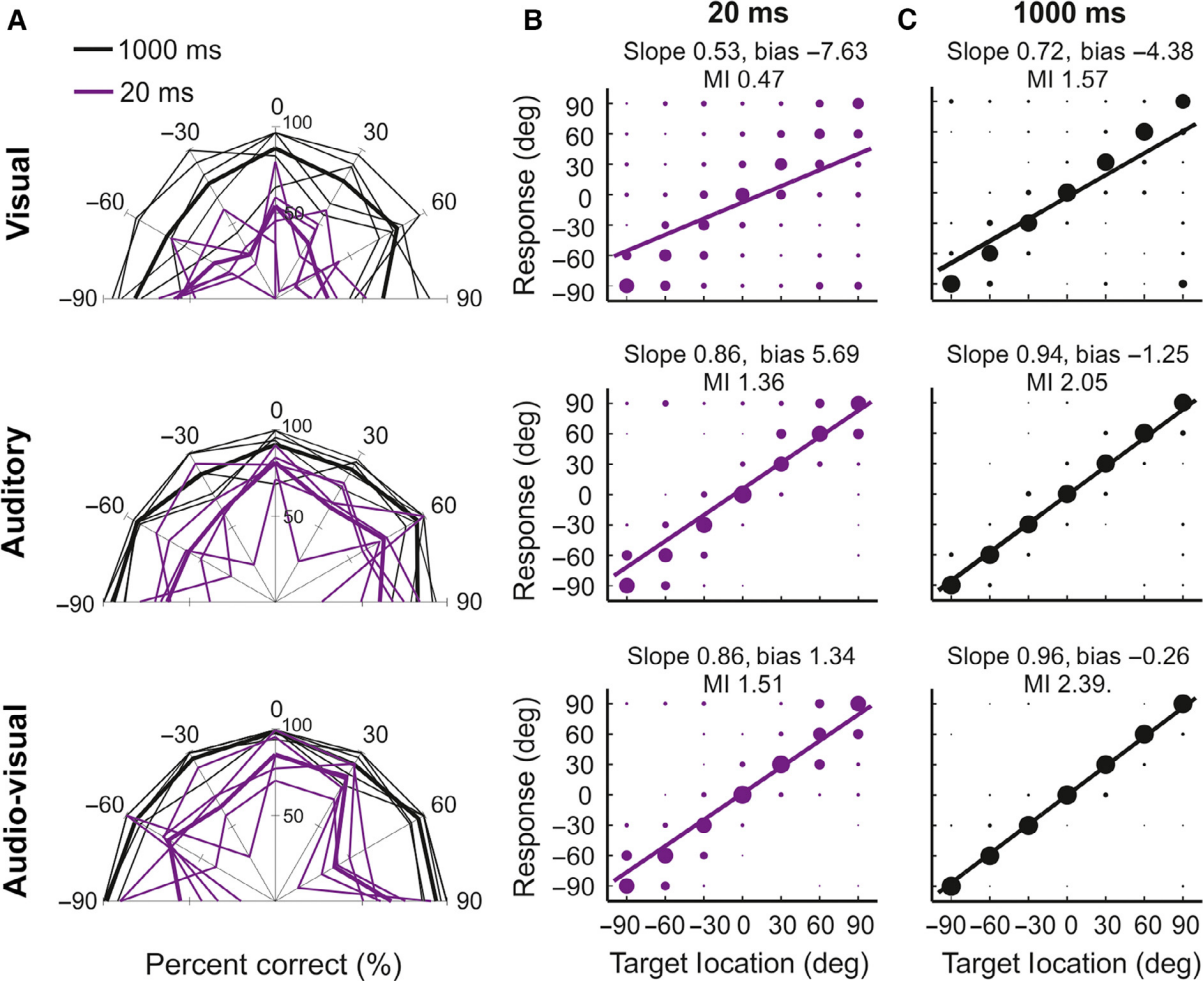
Effects of stimulus modality, duration and location on approach‐to‐target responses. The distribution of approach‐to‐target responses is shown for each target location across different stimulus modalities (top row visual, middle row auditory and bottom row auditory–visual) and durations (20 and 1000 ms). (A) Approach‐to‐target percentage correct scores at each target location for stimulus durations of 20 ms (purple lines) and 1000 ms (black lines). The thin lines indicate the performance of individual animals; thick lines represent the overall mean performance. (B, C) Confusion matrices for these data illustrating the distribution of approach‐to‐target responses. The size of the dots indicates for each stimulus location the proportion of responses made to different target locations. A regression line was fitted (purple and black) to each confusion matrix, using the linear least squares method, and the corresponding slope (gain) and y‐intercept (bias) are shown along with the mutual information (MI) values above the panels (see Table 1 for a complete set of values). [Colour figure can be viewed at wileyonlinelibrary.com].

**Figure 5:**
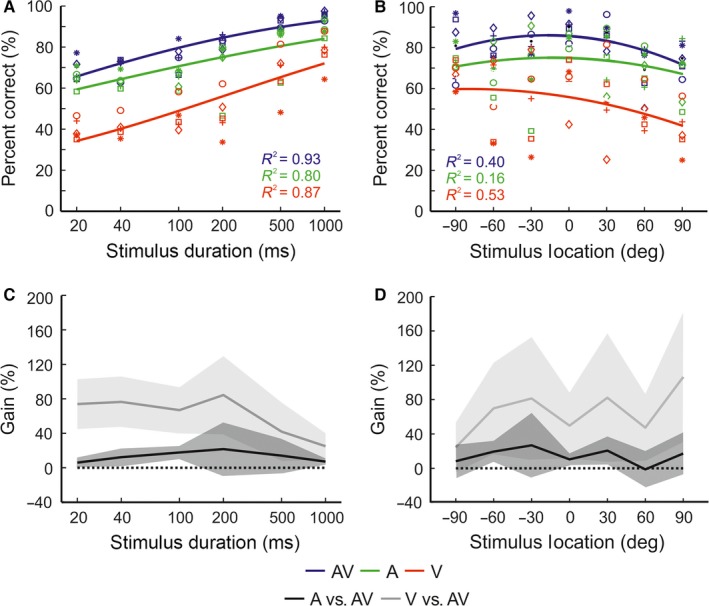
Comparison of approach‐to‐target localization accuracy for unisensory and multisensory targets. Approach‐to‐target percentage correct scores are shown as a function of stimulus duration (A), plotted on a log scale, and stimulus location (B) for auditory–visual (AV, blue), auditory (A, green) and visual (V, red) stimulus targets. Mean percentage correct scores were fitted with a cumulative normal distribution or a polynomial function. The relevant co‐efficient of determination (*R* ^2^) value is indicated for each dataset. Individual animals are represented by different symbols. In contrast to the head‐orienting responses, approach‐to‐target localization accuracy was greater for the auditory–visual targets than for either the auditory or visual targets across all stimulus durations and locations. The multisensory percentage gains (± SD) for the approach‐to‐target responses are shown as a function of stimulus duration (C) and stimulus location (D) for auditory vs. auditory–visual (black) and visual vs. auditory–visual (grey) targets. A positive gain indicates that the mean performance for the multisensory condition is superior to that of the unisensory condition. [Colour figure can be viewed at wileyonlinelibrary.com].

Approach‐to‐target localization accuracy, measured using percentage correct scores, improved across all sensory conditions as stimulus duration was increased (Figs 4 and 5). MI values were calculated and regression lines were fitted (*R* ^2^ range across stimulus durations; visual: 0.17–0.54; auditory: 0.68–0.89; auditory–visual: 0.75–0.94) to quantify the relationship between target and response location, and the slopes and y‐intercepts of these lines compared across stimulus modalities and durations (Table 1). For each stimulus type, both the MI values and slopes tended to increase with increasing stimulus duration, mirroring the improvement in percentage correct scores. As with the head‐orienting responses, the approach‐to‐target responses also became more precise at longer stimulus durations, particularly for the more eccentric target locations (> 30°; Fig. 4B and C).

A clear difference was found in approach‐to‐target localization accuracy for the different stimulus types (Fig. 4 and Table 1). For all stimulus durations, the highest percentage correct scores were obtained for the auditory–visual targets, followed by the auditory targets and then the visual targets. The same pattern was observed by comparing the slopes of the regression lines and the MI between the response and target locations (Table 1).

To investigate the nature of this multisensory enhancement effect, a generalized linear mixed model for Bernoulli‐distributed responses was fitted to the data, to determine the effects of stimulus modality, location and duration on approach‐to‐target percentage correct scores (Fig. 5A and B). For all stimulus duration and location combinations, animals were significantly more likely to make a correct approach‐to‐target response to auditory–visual than to visual stimuli (odds ratio (OR) range across stimulus locations broken down by stimulus duration; 20–40 ms: OR = 2.08–6.20, *t*_(19826)_ = 6.04–14.83, *P* <* *0.001; 100–200 ms: OR = 2.48–7.37, *t*_(19826)_ = 8.16–16.21, *P* <* *0.001; 500–1000 ms: OR = 1.18–8.78, *t*_(19826)_ = 8.50–15.77, *P* <* *0.001). Similarly, they were significantly more likely to respond correctly to auditory–visual than to auditory targets across the majority of stimulus location/duration combinations (OR range across stimulus locations broken down by stimulus duration; 20–40 ms: OR = 1.33–2.31, *t*_(19826)_ = 2.49–6.61, *P < *0.01; 100–200 ms: OR = 1.56–3.06, *t*_(19826)_ = 3.96–8.76, *P < *0.001; 500–1000 ms: OR = 1.32–4.06, *t*_(19826)_ = 2.19–9.32, *P < *0.05), with the exception of 20 ms at ± 90° and 20–500 ms at + 60°, which did not show any significant difference between these stimuli.

To quantify the magnitude of this multisensory facilitation of approach‐to‐target accuracy, multisensory gains were calculated (Fig. 5C and D; see Materials and Methods). Overall, gains were positive when compared against either of the unisensory stimulus conditions and were larger relative to the visual than to the auditory stimuli (auditory–visual vs. visual = 43.17%; auditory–visual vs. auditory = 9.96%). Multisensory gains over the auditory stimuli alone showed no clear correlation with stimulus duration or location, indicating that the improvement in performance was present across the full range of stimulus durations tested (Fig. 5C) and throughout the frontal hemifield (Fig. 5D). However, multisensory gains over the visual stimuli alone were relatively uniform for shorter stimulus durations (≤ 200 ms) only and then declined at longer durations (500 and 1000 ms) (Fig. 5C), whereas no clear differences in performance gain for multisensory vs. visual stimuli were apparent within the frontal hemifield (Fig. 5D).

### Reaction and response times

In addition to measuring localization accuracy, we also analysed head‐orienting reaction times and approach‐to‐target response times to assess whether ferrets showed a redundant signal effect (RSE) (Todd, 1912), according to which they should react and respond more rapidly to paired auditory–visual stimuli than to unisensory stimulation.

**Figure 6:**
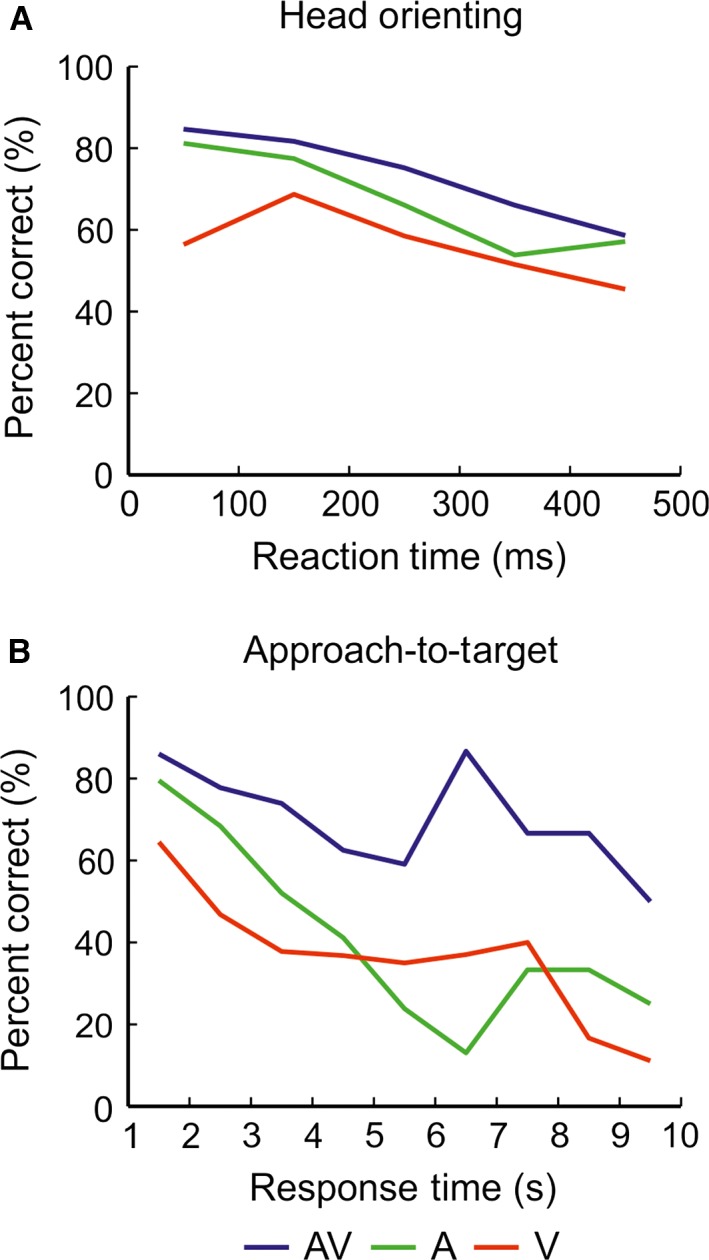
Approach‐to‐target localization accuracy vs. reaction and response times. Approach‐to‐target percentage correct scores are plotted against head‐orienting reaction (A) and approach‐to‐target response (B) times, binned in 100 ms and 1 s intervals, respectively, for auditory–visual (AV, blue), auditory (A, green) and visual (V, red) targets. In general, for all stimulus types incorrect responses were associated with longer reaction and response times than correct responses. [Colour figure can be viewed at wileyonlinelibrary.com].

A correlation between response times and the correctness of approach‐to‐target responses has previously been described in ferrets using a 360° auditory localization task, whereby correct responses were shown to be faster than incorrect responses (Nodal *et al*., 2008). Figure 6 shows a similar trend, regardless of stimulus modality, across the frontal hemifield. To investigate the significance of this effect, two‐dimensional contingency tables (approach‐to‐target response correctness vs. reaction or response times) were constructed separately for each modality. Chi‐squared tests confirmed that head‐orienting reaction times (auditory–visual: $\chi_{8}^{2}$  = 84.73, *P *<* *0.001; auditory: $\chi_{8}^{2}$  = 264.70, *P *<* *0.001; visual: $\chi_{8}^{2}$  = 272.70, *P *<* *0.001) and approach‐to‐target response times (auditory–visual: $\chi_{4}^{2}$  = 38.44, *P *<* *0.001; auditory: $\chi_{4}^{2}$  = 71.16, *P *<* *0.001; visual: $\chi_{4}^{2}$  = 84.78, *P *<* *0.001) were both significantly correlated with the correctness of approach‐to‐target responses.

In view of this result, together with the varying proportion of correct trials observed between stimulus types, incorrect trials were omitted from the following analysis. Furthermore, to prevent any bias due to a possible lack of attention, which might have resulted in abnormally slow responses, reaction times > 500 ms and response times > 10 s were also omitted from the analysis. In total, 1.3% of reaction times and 0.3% of response times were excluded because of these criteria.

**Figure 7:**
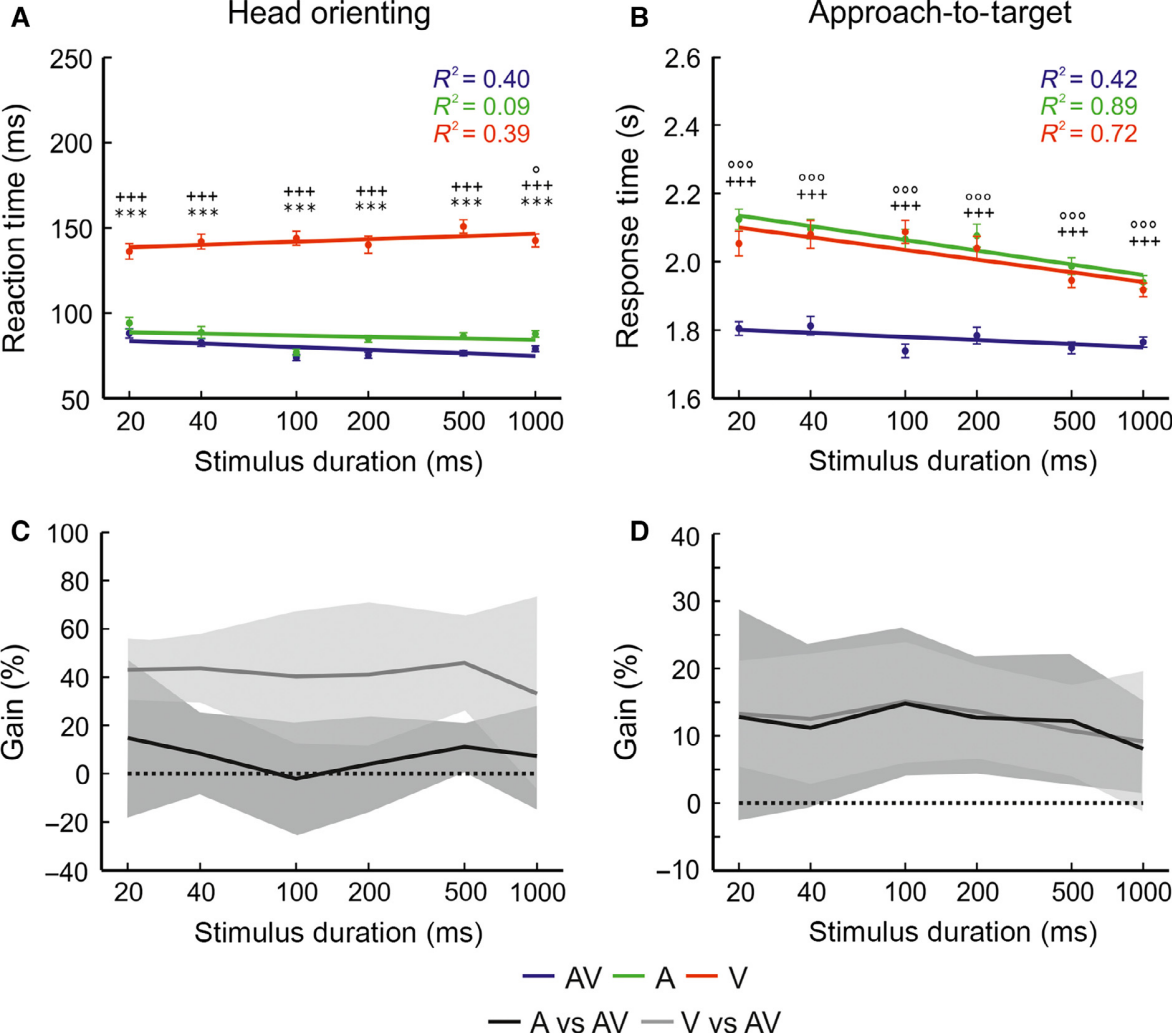
Comparison of head‐orienting reaction and approach‐to‐target response times for unisensory and multisensory targets. Mean (± SEM) head‐orienting reaction times (A) and approach‐to‐target response times (B) are plotted as a function of stimulus duration (on a log scale) for auditory–visual (AV, blue), auditory (A, green) and visual (V, red) targets. For each stimulus type, mean times were fitted with a regression line using the linear least squares method. The relevant co‐efficient of determination (*R* ^2^) value is given for each dataset. Note that reaction and response times were shorter across all stimulus durations for auditory–visual targets than for either auditory or visual targets. Significant differences between stimuli are indicated by the symbols (circles: AV vs. A; crosses: AV vs. V; asterisks: A vs. V and probability value by their number, one: *P *<* *0.05; two: *P *<* *0.01 and three: *P *<* *0.001). (C, D) Mean multisensory gains (± SD) for the head‐orienting reaction (C) and approach‐to‐target response (D) times as a function of stimulus duration for auditory vs. auditory–visual (black) and visual vs. auditory–visual (grey) targets. A positive gain indicates that reaction/response times are shorter for multisensory stimuli than for unisensory stimuli. [Colour figure can be viewed at wileyonlinelibrary.com].

Overall, head‐orienting reaction times (auditory–visual: 77.67 ± 1.01 ms, auditory: 85.70 ± 1.13 ms, visual: 142.14 ± 1.36 ms) and approach‐to‐target response times (auditory–visual: 1.77 ± 0.01 s, auditory: 2.04 ± 0.01 s, visual: 2.02 ± 0.01 s) were shorter for the auditory–visual stimuli than for either of the unisensory stimuli. To investigate the significance of this effect across different stimulus durations, separate anovas were performed on reaction time and response time data (Fig. 7A and B). This analysis identified significant main effects of ferret identity, stimulus modality and stimulus duration for both reaction times (ferret: *F*_(4,10234)_ = 45.54, *P *<* *0.001; modality: *F*_(2,10234)_ = 804.50, *P *<* *0.001; duration: *F*_(5,10234)_ = 4.98, *P *<* *0.01) and response times (ferret: *F*_(4,13700)_ = 547.87, *P *<* *0.001; modality: *F*_(2,13700)_ = 276.94, *P *<* *0.001; duration: *F*_(5,13700)_ = 12.40, *P *<* *0.001). Tukey's multiple comparison *post hoc* tests showed that head‐orienting reaction times were significantly shorter for auditory–visual stimuli than for visual stimuli, whereas, with the exception of the longest stimulus duration, a multisensory advantage was not seen relative to the auditory stimuli (Fig. 7A). In contrast, ferrets took significantly less time to reach auditory–visual targets than either visual or auditory targets (*P *<* *0.001) (Fig. 7B). These differences are supported by the finding that reaction times were significantly shorter for auditory than for visual stimuli at all durations (*P *<* *0.001), whereas for approach‐to‐target response times this was not the case (*P *>* *0.05).

The multisensory influence on the speed of the responses made on correct trials was quantified by calculating the multisensory gain (Fig. 7C and D). Overall, multisensory gains were positive for both reaction times (auditory–visual vs. auditory = 7.24%; auditory–visual vs. visual = 41.19%) and response times (auditory–visual vs. auditory = 11.98%; auditory–visual vs. visual = 12.39%) when compared with either form of unisensory stimulation. In line with the approach‐to‐target performance gains, reaction and response time gains were also relatively constant across stimulus durations.

**Figure 8:**
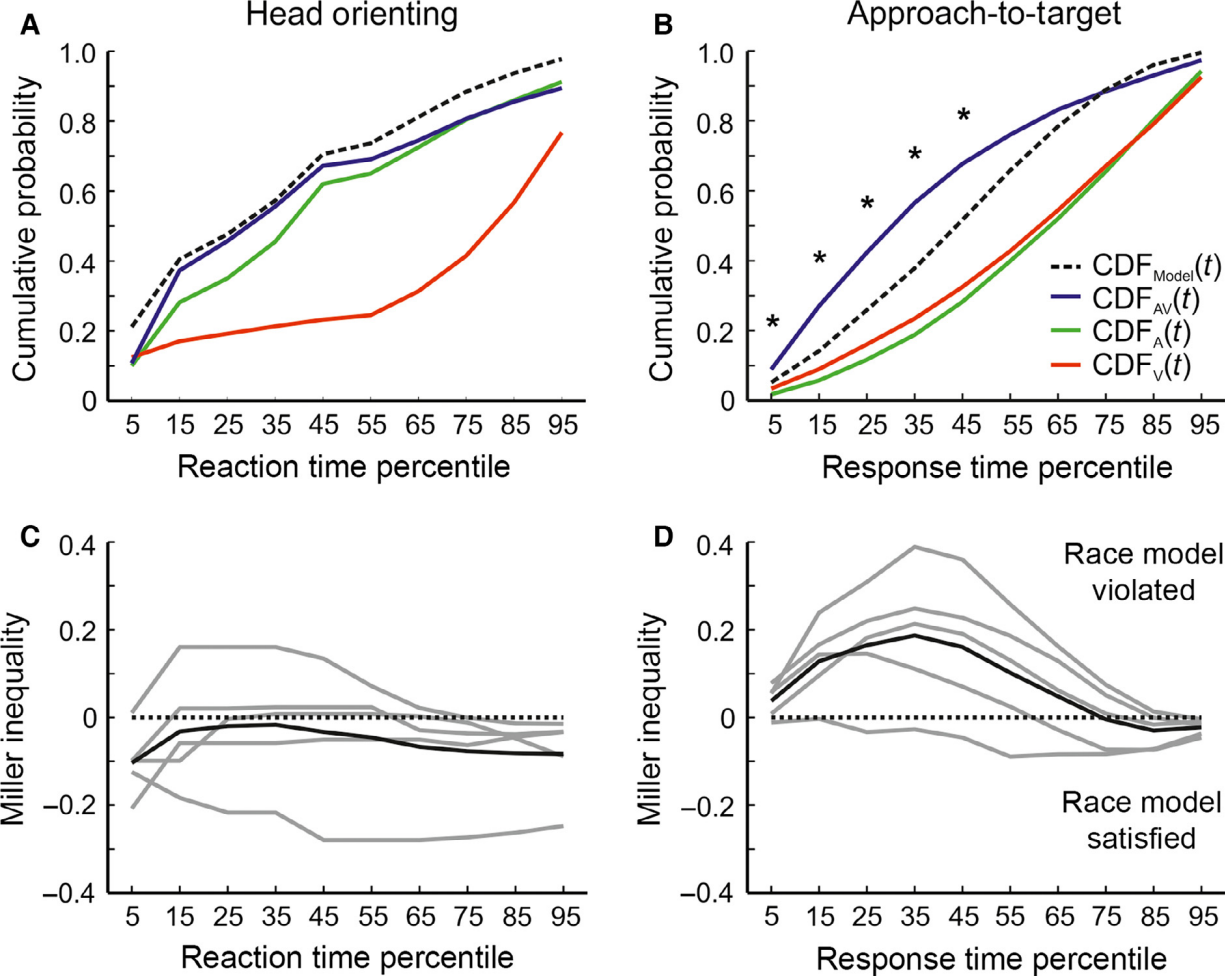
Race model analysis. Group aggregated cumulative probability distribution functions (CDF) of head‐orienting reaction times (A) and approach‐to‐target response times (B), displayed for auditory–visual (AV, blue), auditory (A, green) and visual (V, red) targets and for the race model (black). In the case of the approach‐to‐target data, the model can be rejected as the auditory–visual CDF (blue solid line) exceeds the modelled CDF (black dashed line), with significant differences across response time percentiles marked by asterisks (*, *P* < 0.05). (C, D) Results of Miller's inequality (CP_(t)_ _AV_ – CP_(t)Model_) test of the race model (see Methods for details) for head‐orienting reaction times (C) and approach‐to‐target response time (D): positive values indicate violation of the model and negative values its satisfaction. Values for individual animals are shown by the grey lines; group aggregated values are shown by the black lines. [Colour figure can be viewed at wileyonlinelibrary.com].

Finally, data were subjected to race model inequality analysis (Raab, 1962) to investigate the basis for the observed RSE (Fig. 8). It is possible to derive the maximum multisensory facilitation of reaction and response times that can be expected given a race scenario by taking the sum of the auditory and visual reaction (or response) time cumulative distribution functions (CDF, see Materials and methods). The model is violated if, at any time, the observed auditory–visual CDF exceeds the modelled CDF, which by default implies the existence of a signal integration mechanism (Miller, 1982).

Analysis of approach‐to‐target response times showed that the model was consistently violated over the first seven deciles (Fig. 8B and D), suggesting that multisensory integration in the brain could be responsible for the faster responses. As might be expected, significant violations were reliably observed at the lower deciles (the 1st to the 5th), as summation of auditory and visual inputs would most likely result in faster times by reaching the response threshold earlier than either input in isolation. On the other hand, analysis of reaction times showed no violation of the model (Fig. 8A and C), implying that the multisensory facilitation of reaction times could be explained in terms of probability summation alone.

## Discussion

In this study, we characterized the ability of ferrets to localize unisensory and multisensory stimuli of different durations, by measuring both their initial head‐orienting and subsequent approach‐to‐target behaviour. Approach‐to‐target localization responses were more accurate and faster to spatially congruent auditory–visual stimuli throughout the frontal hemifield than to either visual or auditory stimuli presented in isolation. Race model inequality analysis of the response times indicated that this multisensory advantage reflects neural integration of the cues available to each sensory modality. Conversely, while head‐orienting responses to auditory–visual stimuli were more accurate and faster than those made to visual targets, this was not the case when they were compared to the responses made to auditory targets, suggesting that these movements were guided principally by sound alone. Moreover, auditory–visual head‐orienting reaction times could be accounted for by probability summation of the unisensory responses, implying that different processes are involved in mediating the effects of multisensory stimulation on these two measures of localization behaviour.

Vision generally provides higher‐resolution spatial information than audition about distant objects (DeValois & DeValois, 1990; Brown & May, 2005). Indeed, ferrets can resolve drifting sine‐wave gratings with a spatial frequency of 0.5 cycles per degree (von Melchner *et al*., 2000), whereas their minimum audible angles are ~ 10° at the midline (Kavanagh & Kelly, 1987; King & Parsons, 1999). Nevertheless, we found that ferrets localized single presentations of broadband noise more accurately than light flashes of equivalent duration from LEDs. This was observed in both the head‐orienting and approach‐to‐target data and across all stimulus durations and locations, and therefore cannot be attributed to the most eccentric visual stimuli falling outside the field of view. This is unlikely to be due to the way they were trained, as all animals were trained initially using auditory–visual stimuli and were not tested until stable criterion levels of performance were achieved with each stimulus type. Although a guiding role for visual cues in the plasticity of auditory spatial processing has been demonstrated during development (King *et al*., 1988) in this species, it is possible that the orienting behaviour of ferrets naturally relies more on their hearing than their sight. This is consistent with our finding that, for the stationary stimuli used in this study, the latency and accuracy of their head turns to spatially congruent auditory–visual stimuli were not significantly different from those made to sound alone.

We measured head movements following stimulus presentation as a metric for assessing the initial orienting response of the ferrets. The head movements directed towards visual and auditory targets had a comparable range of amplitudes and latencies to those described in cats (Ruhland *et al*., 2013), with reaction times ~ 50 ms shorter when the animals localized a sound source. We found that the relationship between final head bearing and target location was similar for the different stimulus types, with the notable exception that head movements to the most peripheral visual stimuli were smaller than those made to auditory or paired auditory–visual targets. Again, this is unlikely to be due to the animals not seeing the most eccentric visual targets (which extended out to ± 90°), as the visual field in ferrets covers at least 110° (King & Hutchings, 1987).

This stimulus‐dependent difference in head orienting could reflect a smaller contribution of head movements (as opposed to eye‐in‐head movements) to the gaze shifts made to visual than to auditory stimuli, as has been reported in cats (Ruhland *et al*., 2013). However, we think this is unlikely as the ferret eye contains a prominent visual streak and a much lower variation in retinal ganglion cell density across the retina (Stone, 1965; Henderson, 1985), raising the possibility that this species relies less on eye movements than other carnivores. Indeed, the lateral rectus muscle, which is responsible for abduction of the eye, has a much slower twitch contraction time and is innervated by relatively few abducens nucleus motoneurons compared to cats (Bishop *et al*., 1985). Moreover, the approach‐to‐target responses showed a comparable dependence on stimulus location and duration and were also consistently more accurate for auditory than for visual stimuli, indicating that head movements alone provide an appropriate measure of localization performance. Although our ferrets were able to localize 20‐ms light flashes from an LED positioned 70 cm away at well above chance levels, the noise bursts presumably provided more salient cues at all durations tested.

In humans, combined eye–head gaze shifts tend to have significantly shorter latencies than the shortest unisensory reaction time across a range of eccentricities (Goldring *et al*., 1996), whereas we observed no difference for most target locations between the head movements made to auditory and auditory–visual targets. Consequently, the multisensory gain was small and could be accounted for by the race model, implying that the orienting movements are triggered by the brain's response to the auditory stimulus rather than the later response to the visual stimulus. However, the relative metrics of eye and head movements can vary with stimulus modality and target location in complex ways (Goldring *et al*., 1996; Ruhland *et al*., 2013), so we cannot rule out the possibility that a clearer multisensory advantage would have been observed had eye–head gaze shifts been measured.

In contrast, our measurements of approach‐to‐target behaviour provided robust evidence for neural integration of auditory and visual inputs. Combining auditory and visual stimuli at the same location has been shown to improve localization accuracy in humans (Alais & Burr, 2004; Odegaard *et al*., 2015) and cats (Stein *et al*., 1989; Gingras *et al*., 2009). Although these studies did not report response times, we found that ferrets not only localized auditory–visual stimuli more accurately than either modality by itself, but they also made significantly faster responses. Indeed, the measured responses times were shorter than those predicted by the race model, indicating an effect of multisensory convergence on the neural processing of spatial information.

A number of brain regions have been implicated in the integration of auditory and visual cues. Spatial information from different sensory modalities is combined in the superior colliculus (SC), which plays a key role in the generation of eye–head gaze shifts (Freedman & Sparks, 1997; Bell *et al*., 2005; Gandhi & Katnani, 2011). The ferret SC conforms to the general vertebrate plan whereby visual and auditory representations are organized topographically to form overlapping maps of sensory space (King & Hutchings, 1987), with neurons in the deeper layers of this mid‐brain nucleus often responsive to stimuli in more than one sensory modality (King & Schnupp, 2000; Meredith *et al*., 2000). More extensive studies in other species have shown that the activity of these multisensory neurons can be altered when different stimuli are combined, with the strongest response enhancement occurring for visual and auditory stimuli that are roughly coincident in space and time (King & Palmer, 1985; Meredith & Stein, 1986; Meredith *et al*., 1987; Wallace *et al*., 1996). Moreover, the ability of cats to orient towards and approach visual and auditory targets in the frontal hemifield is impaired following reversible inactivation of the superficial and intermediate layers of the SC, respectively (Lomber *et al*., 2001), while excitotoxic lesions of the latter results in a more persistent loss of the modulatory effect of one stimulus modality on another in this task (Burnett *et al*., 2004).

However, the SC does not act alone in mediating cross‐modal influences on localization behaviour, as these effects are also reduced in cats following inactivation of the anterior ectosylvian sulcus, a multisensory cortical region that innervates the SC (Wilkinson *et al*., 1996; Bajo *et al*., 2010). It is also possible that other cortical regions are involved as there is now extensive evidence for multisensory convergence and interactions in primary and non‐primary sensory areas (e.g. Falchier *et al*., 2002; Rockland & Ojima, 2003; Bizley *et al*., 2007; Lakatos *et al*., 2007; Hall & Lomber, 2008; Kayser *et al*., 2010; Henschke *et al*., 2015). The auditory cortex is likely to be particularly important in this respect as spatially coincident visual cues can enhance spatial processing by auditory cortical neurons in ferrets (Bizley & King, 2008), whereas studies of ventriloquism in humans (Bonath *et al*., 2007) and monkeys (Recanzone, 1998) have implicated the auditory cortex in the shift in the perception of auditory space produced by presenting spatially discrepant auditory–visual stimuli. Previous work in our laboratory has shown that silencing the primary auditory cortical fields in ferrets impairs approach‐to‐target localization accuracy when short‐duration noise bursts are used as a stimulus, without affecting head‐orienting behaviour (Nodal *et al*., 2010, 2012), suggesting that the neural circuits involved in guiding these responses may not be the same. This is supported by our finding that improvements in the speed and accuracy of auditory localization by the provision of spatially coincident visual cues were only observed when the animals had to select which target to approach to receive a reward, but not when they merely turned towards the target.

It is also possible that the multisensory interactions that we observed involve the visual cortex. Projections to visual areas V1 and V2 from the auditory cortex have been described in monkeys (Falchier *et al*., 2002; Rockland & Ojima, 2003) and cats (Hall & Lomber, 2008). Intriguingly, these inputs principally target the peripheral visual field representations and, in cats, originate from the posterior auditory field, which has been shown to play a particularly significant role in sound localization (Lomber & Malhotra, 2008). This led Hall & Lomber (2008) to suggest that these projections may serve to link cortical areas responsible for auditory and visual localization, potentially providing an anatomical substrate for auditory cues to improve the localization of peripheral visual targets, where spatial precision and accuracy are lowest. However, although we did indeed find that the difference between the final head bearings for visual and auditory–visual targets increased with stimulus eccentricity, this could be accounted for by the responses to the auditory targets. Moreover, the multisensory gain in the accuracy of approach‐to‐target responses was relatively constant throughout the frontal hemifield and not restricted to peripheral targets. Further evidence against an involvement of visual cortex in this process comes from the finding that presentation of spatiotemporally coincident visual stimuli in the blind hemifield of hemianopic patients still improves auditory localization accuracy (Leo *et al*., 2008), while suppression of occipital cortex by repetitive transcranial magnetic stimulation in healthy subjects does not affect the visual enhancement of auditory localization (Bertini *et al*., 2010).

Elucidation of the neural circuits responsible for multisensory spatial processing will require not only the application of more refined methods for manipulating activity in specific neuronal populations (e.g. Olcese *et al*., 2013; Wasserman *et al*., 2015), but also measurements of the activity of those neurons during behavioural tasks. Recordings from the brain during orienting behaviour have focussed predominantly on the SC, posterior parietal cortex and, to a lesser extent, the auditory cortex and other parts of the auditory pathway, and have highlighted the importance of remapping sensory representations that are used to guide movements into common reference frames (reviewed in Maier & Groh, 2009). But whereas the SC contains topographically aligned maps of sensory space, it is less clear how visual inputs are coordinated with auditory representations, at least in early levels of cortical processing that are tonotopically organized. Simultaneous recordings while animals carry out the auditory–visual localization task described in this study should provide further insight into the functional significance of multisensory convergence in the auditory cortex and surrounding areas and into the way spatial information is representation there.

## Conflict of interest

The authors have no conflict of interest to declare.
